# The Research Agenda for Perinatal Innovation and Digital Health Project: Human-Centered Approach to Multipartner Research Agenda Codevelopment

**DOI:** 10.2196/60825

**Published:** 2025-01-30

**Authors:** Haneen Amhaz, Sally Xuanping Chen, Amanee Elchehimi, Kylin Jialin Han, Jade Morales Gil, Lu Yao, Marianne Vidler, Kathryn Berry-Einarson, Kathryn Dewar, May Tuason, Nicole Prestley, Quynh Doan, Tibor van Rooij, Tina Costa, Gina Ogilvie, Beth A Payne

**Affiliations:** 1 Women's Health Research Institute Vancouver, BC Canada; 2 BC Children's Hospital Research Institute Vancouver, BC Canada; 3 Faculty of Health Sciences Simon Fraser University Burnaby, BC Canada; 4 Department of Obstetrics and Gynaecology Faculty of Medicine The University of British Columbia Vancouver, BC Canada; 5 Quality and Research Perinatal Services BC Provincial Health Services Authority Vancouver, BC Canada; 6 Provincial Digital Health and Information Services Provincial Health Services Authority Vancouver, BC Canada; 7 BC Ministry of Health Victoria, BC Canada; 8 Department of Pediatrics Faculty of Medicine The University of British Columbia Vancouver, BC Canada; 9 Department of Computer Science Faculty of Science The University of British Columbia Vancouver, BC Canada; 10 School of Population and Public Health Faculty of Medicine The University of British Columbia Vancouver, BC Canada

**Keywords:** digital health, co-design, digital strategy, human-centered design, eHealth, cocreation, codevelopment, perinatal intervention, quality of care, digital tools, pregnancy, patient autonomy, patient support, mobile phone

## Abstract

**Background:**

Digital health innovations provide an opportunity to improve access to care, information, and quality of care during the perinatal period, a critical period of health for mothers and infants. However, research to develop perinatal digital health solutions needs to be informed by actual patient and health system needs in order to optimize implementation, adoption, and sustainability.

**Objective:**

Our aim was to co-design a research agenda with defined research priorities that reflected health system realities and patient needs.

**Methods:**

Co-design of the research agenda involved a series of activities: (1) review of the provincial Digital Health Strategy and Maternity Services Strategy to identify relevant health system priorities, (2) anonymous survey targeting perinatal care providers to ascertain their current use and perceived need for digital tools, (3) engagement meetings using human-centered design methods with multilingual patients who are currently or recently pregnant to understand their health experiences and needs, and (4) a workshop that brought together patients and other project partners to prioritize identified challenges and opportunities for perinatal digital health in a set of research questions. These questions were grouped into themes using a deductive analysis approach starting with current BC Digital Health Strategy guiding principles.

**Results:**

Between September 15, 2022, and August 31, 2023, we engaged with more than 150 perinatal health care providers, researchers, and health system stakeholders and a patient advisory group of women who were recently pregnant to understand the perceived needs and priorities for digital innovation in perinatal care in British Columbia, Canada. As a combined group, partners were able to define 12 priority research questions in 3 themes. The themes prioritized are digital innovation for (1) patient autonomy and support, (2) standardized educational resources for patients and providers, and (3) improved access to health information.

**Conclusions:**

Our research agenda highlights the needs for perinatal digital health research to support improvements in the quality of care in British Columbia. By using a human-centered design approach, we were able to co-design research priorities that are meaningful to patients and health system stakeholders. The identified priority research questions are merely a stepping stone in the research process and now need to be actioned by research teams and health systems partners.

## Introduction

The digital health sector continues to expand after being an integral part of health care delivery during the COVID-19 pandemic, resulting in a growing interest in digital health research and innovation. Digital health provides an opportunity to reduce health disparities by addressing barriers and facilitators to health care access and improving the health and lifestyle of those who use it [1]. Digital innovation is also essential to offering value-based health care, which involves changes in health systems that are measured by improvement in patient outcomes per unit health care expenditure [2]. Core to achieving improvements in value-based care is establishing an understanding of what value is based on identifying outcomes that matter to patients themselves.

Human-centered design (HCD) is an approach to problem-solving that can be used to increase value in health care by focusing on problems and outcomes of interest to patients. HCD also has a role in improving health equity [3,4]. Core characteristics of an HCD approach include an iterative process of empathy and discovery to identify the core problem and then systematic brainstorming of solutions where the human experiencing the problem plays a central role [5]. The HCD approach goes beyond simply working with the end user to focus on their interaction with the technology and considers the broader social context in which the technology is used and other partners who may indirectly be impacted by the use of the technology [4,5]. The result of working in partnership with people under these assumptions using the HCD approach is meant to be a product or service that meets real human needs and reflects their values. This approach has been used to solve complex health care challenges, such as improving pediatric patient experience in acute care [6] and increasing adherence to self-care practices for chronic conditions [7,8]. In a perinatal health setting, these methods have been used to develop digital interventions for gestational diabetes [9].

The perinatal period encompasses a person’s experiences from conception to the first year after delivery including pregnancies that end in miscarriage or stillbirths. The majority of patients receiving perinatal care in British Columbia has access to a mobile device and the internet and are increasingly looking to digital technologies to inform and support their care [10,11]. Using digital technology in health care can improve the quality of care and health outcomes for patients and infants during this early stage of life. The use of digital health tools in perinatal care has already been shown to result in lower and healthier gestational weight, lower smoking rates, increased physical activity, and lower rates of maternal and infant morbidity (eg, fewer preterm births, gestational diabetes, and pre-eclampsia), leading to fewer planned and unplanned visits to health care facilities [1,12-14]. Digital health has also been demonstrated to reduce the practitioners’ workload as well as relieve some of the resource strains on an overburdened system [15]. Due to the vast potentiality of digital health and opportunities for further innovation, it is important to engage with patients who have received perinatal care and care providers as end users to identify emerging priorities for future innovations and research that are informed by actual needs [16]. By identifying and responding to real patient and care provider needs, we can optimize the implementation, adoption, and sustainability of perinatal digital health solutions, create value, and ensure existing health inequities are not exacerbated [16,17].

In this paper, we report on the Research Agenda for Perinatal Innovation and Digital Health (RAPID) project [18], which was led by the BC Children’s Hospital Research Institute and Women’s Health Research Institute in partnership with the Provincial Health Services Authority (PHSA) and Perinatal Services BC (PSBC) with the aim of codeveloping research priorities with patients and care providers.

## Methods

### Ethical Considerations

This project was run as a knowledge translation initiative, and no research ethics board approval was required. The engagement activities conducted with partners were classified as quality improvement activities rather than systematic investigations to advance knowledge. Based on these distinctions, our institutional research ethics board determined that the project falls outside of the scope of research subject to ethics review, and a waiver was granted. All workshop and survey participants consented to the anonymous collection of their opinions. Members of our patient advisory committee were compensated hourly according to local standards to reflect their equal standing to research team members within the project.

### Project Design and Setting

The patient and health system partners with whom we worked were partners in the research priority setting through co-design and were compensated for their time. All final workshop participants provided consent to the collection of their opinions and views during the workshop and through the follow-up survey.

The objectives of the RAPID project were to facilitate activities that bring key partners and existing knowledge together to (1) identify gaps in the perinatal continuum of care that can be addressed with digital innovation to improve patient and family health and (2) define current barriers to optimal adoption of digital health interventions for perinatal health care from the patient and health system perspectives. This knowledge was then used to define research priorities and codevelop research questions to motivate digital innovation informed by care needs and addressing barriers.

### Recruitment of Partners and Governance

Following guidance outlined by Concannon et al [19], we developed an engagement plan for our activities that describes an analysis of which partner groups should be involved, their roles and modes of participation, and sampling strategies (ie, snowball sampling). Partners to include in engagement activities were grouped as (1) people with lived experience of perinatal care who were pregnant or recently delivered a baby; (2) perinatal health care providers including nurses, midwives, physicians, and other allied health; and (3) health care decision makers who are involved with the design or delivery of perinatal health care in British Columbia.

This project was governed by two primary groups: (1) a steering committee and (2) a patient advisory committee (PAC). Using snowball sampling, starting with a core group of experts at BC Children Hospital Research Institute and Women’s Health Research Institute, we identified key partners with expertise in research, innovation, and implementation processes related to perinatal, maternal, or digital health services to form the steering committee. The core group was involved from the initiation of the project including during the development of the funding proposal. This core group included 4 health system decision makers as research users and 4 researchers. Additional partners identified for the steering committee included 2 clinical care providers and 2 additional researchers. The final steering committee included 5 researchers, 3 health care providers, and 4 health system decision makers. The purpose of the steering committee was to oversee project objectives and align efforts to each of their organizations’ known digital health priorities and ongoing work.

Patient partners for the advisory committee were recruited through social media and the local hospital patient engagement office. The criteria for participation were (1) multilingual and (2) pregnant or a caregiver of a child younger than 2 years of age. There were 15 respondents to the recruitment advertisements, 9 of whom responded to our invitation to complete a 15-minute informal interview so we could learn more about their background, and they could also learn more about our project. After the interviews, the steering committee was able to select 4 participants who were willing and able to commit the time required by the project and who had diverse perinatal experiences (based on type of care provider and mode of delivery), geographic location in British Columbia, and backgrounds (language spoken and profession). The PAC met separately with only 4 members of the study team and steering committee during the initial activities of the project. This was done to create a safe space that would limit the impact of known health system hierarchies and preserve the authentic patient voice in the identification of perinatal health system pain points and the development of initial insights related to these pain points [20]. Once initial insights were defined with the patient advisors, all partners were brought together in a final workshop to collaborate and build consensus on research priorities as described in more detail below.

### Engagement Activities

A series of engagement activities with each distinct group of partners was completed before a final workshop that brought all partners and results together to reach a consensus on final research priorities.

#### Perinatal Care providers

Perinatal care providers across British Columbia including physicians, midwives, nurses, and allied health were invited to share, rate, and explore their thoughts on digital perinatal health using a ThoughtExchange (version 6.4; Fulcrum Management Solutions Ltd). ThoughtExchange is a web-based survey tool that allows participants to share their thoughts in response to a prompt, which can be anonymously viewed and ranked by other participants. The platform then creates weighted rankings of the thoughts on the prompted topic to show participant priorities. The care providers were asked to respond to the following prompt: “What digital health tools are most valuable to your delivery of perinatal health care currently, and what do you think are the greatest areas of opportunity for digital innovation going forward?”* *The survey was promoted through all British Columbia health authority internal staff newsletters and through member email lists at Doctors of BC; Family Doctors of BC; and University of British Columbia Faculty of Medicine alumni, School of Midwifery, Departments of Obstetrics and Gynaecology, Pediatrics and Family Medicine. Three rounds of promotion were completed. Once the advertised deadline passed, the highest-ranked thoughts were reviewed by 2 research members of the steering committee (BAP and SXC), and key messages were developed through an inductive approach. Basic descriptive statistics were used to analyze the ranking of thoughts.

#### Health System Partners

Steering committee members from the British Columbia Ministry of Health (MoH), the PHSA, and the PSBC were each asked to review their organizations’ recently published reports outlining their strategies for addressing current gaps in health. The reports reviewed were the BC Digital Health Strategy (BCDHS) [21] and the PSBC Maternity Services Strategy (MSS). Access to the MSS was provided through confidential personal communication with PSBC Director Robert Finch. These reports were presented at a steering committee meeting within the first 3 months of the project to identify priorities and alignments specific to perinatal digital health within these documents. The steering committee members from each organization outlined ongoing digital innovation projects in perinatal care and priorities specific to digital health solutions for improving perinatal care. Each report, postpresentation, was summarized in lay terms to highlight priorities. These summaries were reviewed and edited by the health system partners to ensure that summaries were comprehensive, not leaving out key information.

#### Patients

A series of four 90-minute PAC meetings were held and recorded over Zoom (Zoom Video Communications). They served to ascertain current digital health use and perceived need for new innovation as well as to inform workshop development from a patient perspective. The meetings included a series of HCD and user-experience research activities, participatory discussions, and postmeeting homework that typically took an hour to complete. The first meeting served as an introduction to all members; the following 2 meetings were developed to allow patients to share their experiences and understand how they interact with the health care system in order to identify gaps, common touchpoints with health care systems and services, and needs (Table 1). For the final meeting, the PAC was provided the lay summaries from the engagement with other partners prior to the meeting. They were asked to review these and share their reflections during the meeting. In addition, they were asked to develop insight statements that identify a need or gap. All meetings were facilitated by the core research team consisting of BAP, SXC, HA, and MV, who were also steering committee members. Activities were carried out on Miro (version 2.0; Miro), a web-based collaborative workspace. Meeting notes were collected and circulated to all participants for review and input. After each meeting, study team members also recorded reflective notes.

**Table 1:** Patient engagement meeting plan.

Session number	Objective	Presession activity	Activities
1	Establish an environment where patients feel safe and comfortable sharing their experiences	N/A^a^	Ice breaker Introduction to digital health Empathy mapping activity
2	Gather patients’ perspectives and experiences in the health system during pregnancy and after birth	Complete an empathy map [22] and a 30-minute reflection on their experiences in perinatal care	Share empathy map and experiences Introduce user journey activity
3	Identify health system touchpoints and potential areas with opportunities for improvement	Complete a user journey [23] Reflect on fellow participants’ empathy maps and reflections	Open discussion regarding user journey and experiences Introduce insightful statements
4	Prepare for workshops with patients	Review summary of ThoughtExchange and health systems’ report summaries Insightful statements worksheet [24]	Comment on summary results Go over insightful statements Plan workshop

^a^N/A: not applicable.

#### Final Consensus Workshop and Analysis

A final 90-minute workshop was held to bring together all the partners and interested parties to share and align on challenges and priorities collected from earlier engagement sessions. This workshop was composed of both existing members of each partner group: patients, clinical care providers, researchers and health system decision makers as well as additional participants recruited through snowball sampling.

**Figure 1:**
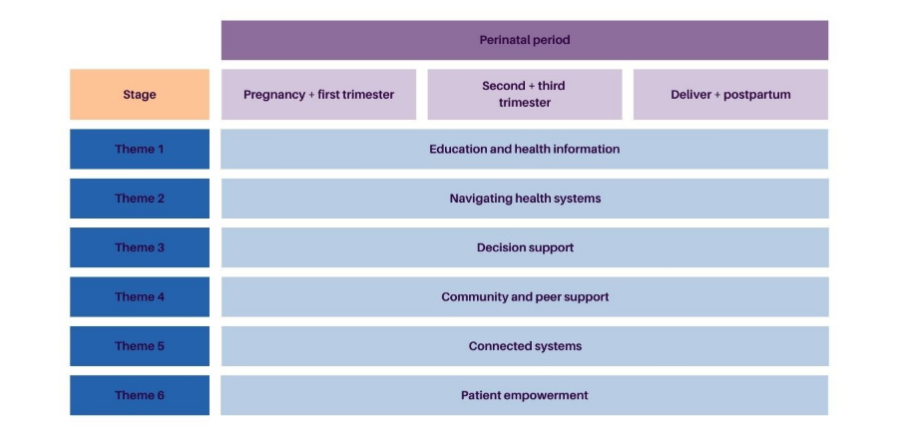
Workshop groups and themes.

In preparation for the workshop, the core study team (BAP, SXC, HA, and MV) reviewed insight statements from the patients, and using a combination of deductive (using the guiding principles of the BCDHS as a starting point) and inductive analysis, placed these into overarching and emerging themes and then further grouped statements based on relevance to a stage of the perinatal period as described in Figure 1. This grouping was done to make the review and brainstorming session more feasible to complete within our limited 90-minute time frame. Research representatives from the steering committee, BAP and MV, developed 2 example research questions for each thematic area to prime the workshop discussion. These questions were developed to prompt discussion during the workshop, considering many participants do not have a background in research.

Attendees were divided into the 3 perinatal stage–based groups based on their expertise. For example, an attendee who is a neonatologist was assigned to the delivery+postpartum group. Each group contained at least 1 member from each of the partner types in order to facilitate collaboration across groups and consensus building. Each group was asked to reflect on and revise the provided insight statements and example research questions as well as develop their own research questions in order to identify research priorities based on their knowledge and experience. At the end of the session, each group was asked to identify 3-5 priority research questions from their brainstorming exercise. Each group then presented their priority research questions to the full workshop, and collectively, all attendees further discussed and ranked the research questions in order of priority.

After the workshop, the research team reviewed meeting recordings, notes, and research question rankings to finalize a list of priority research questions that reflected workshop consensus. These research questions were then reviewed a final time by the PAC to ensure patient needs were at the forefront. This final list of research questions was converted and deployed as a REDCap (Research Electronic Data Capture; Vanderbilt University) survey to all workshop attendees. Respondents were asked to select 3 research questions they believed to be of the highest priority for perinatal care. Survey rankings were summarized descriptively to identify priorities from within the defined research questions.

## Results

Engagement activities occurred between September 15, 2022, and August 31, 2023. The steering committee met monthly during this time to review progress and plan adjustments to the strategy, as new knowledge was developed.

### Thought Exchange With Clinical Care Providers

A total of 125 perinatal health care provider participants responded, 102 of whom shared what provincial health authority they were affiliated with. British Columbia is split by geographic regions into 5 regional health authorities with an additional province-wide authority focused on Indigenous health and the PHSA that provides oversight for all regions. Most participants came from PHSA (n=25, 24.5%), Fraser Health (n=19, 18.6%), Vancouver Coastal Health (n=16, 15.7%), or Island Health (n=16, 15.7%). There were no participants from the First Nations Health Authority, and 7 participants did not affiliate with any of British Columbia’s 7 health authorities.

Of the 125 participants, 55 responded with 80 different thoughts; 51 participants rated the thoughts of their colleagues, resulting in a total of 1108 ratings. Analysis of the thoughts based on the built-in functions of the ThoughtExchange platform identified the highest-ranked themes, which were grouped and summarized. Five key takeaways were identified under the themes of health education and connected systems from the ThoughtExchange:

1. There is an urgent need to create more inclusive, responsive, and accessible digital health tools. The end user (patient or health care provider) must be involved in the design of these tools to make sure that they are useful and can solve the right problems.
2. Among all current digital health tools shared, “Zoom,” which is a tool that allows videos and remote health visits, and the “Hyperbilirubinemia risk assessment tool,” which is a way for doctors to check if a baby is at risk of developing a condition where their skin and eyes turn yellow, are the highest ranked. “Up to Date,” a web-based platform that keeps clinicians up-to-date with the latest medical research and guidelines (the United States only), was mentioned but ranked lower. A tool like “Up to Date” that provides the latest guidelines specific to Canada was identified as a current need to support high-quality care.
3. Important clinical areas that were identified as being priority topics for digital innovation were postpartum lactation support and mental health services. There is a demand for innovation in these areas to be accessible to individuals residing in rural regions.
4. A 2-way communication system is needed to support sharing health information across the province and between patients and providers; this means patients should have equal access to their electronic medical records as their health care providers. Patients should also be able to freely share their information between various levels of health care units at their own discretion.
5. Provincially standardized, free, evidence-based, and user-friendly prenatal patient education was identified as a need that could be solved with digital innovation. Accessible prenatal education would support high-quality perinatal care for all patients in British Columbia.

### Health System Partner Engagement

A review of the MoH and PSBC digital health reports and strategies with health system partners in the steering committee meetings identified opportunities where perinatal innovation could be used to improve the quality of the health care system in British Columbia.

The MoH report, BCDHS, identified the following gaps in care across British Columbia: lack of coordination between health care services, poor communication systems, and inadequate follow-up. Therefore, their digital health priorities include (1) empowering patients through participation in their health journey (eg, inclusive, trusted, and equitable health content provided digitally), (2) increasing the capacity for providers to deliver high-quality care (eg, improving digital literacy and promoting the use of digital tools), (3) establishing a connected health system that allows for the sharing of data (eg, interoperability across health authorities and provider level and collaborating on clinical solutions), and (4) infrastructure and business processes streamlined for efficiency (eg, modernizing British Columbia’s health supply chain, data flow, asset tracking, and analytics). To accommodate these strategies and goals, PSBC is working on developing a digital platform that acts as a central place for patient information. Based on the identified gaps, strategies, and needed tools, three potential areas for research were identified: (1) What is the best way to use telecommunication and information technologies to provide health care services during pregnancy and childbirth remotely? (2) What is the best design and implementation strategy to enable a shared electronic antenatal health record that can be accessed by any health care provider and patient during pregnancy? (3) Can we validate the use of wearable devices that can track and monitor health data during pregnancy and childbirth (ie, remote blood pressure for hypertension in pregnancy and blood glucose monitoring for gestational diabetes)?

PSBC’s MSS report focuses particularly on addressing modifiable factors such as maternal weight and nutrition, substance use, mental health, diabetes, infant sleep, and breastfeeding. They currently have a series of projects in progress, and those specific to digital health solutions include a personalizable information and tool resource hub for providers and patients, a single source of data that can be shared across multiple systems, leveraging access to clinical data for quality improvement, allowing for enhanced digital training opportunities, increasing access to telemedicine care, and updated screening protocols and quality control. Based on these approaches five areas for future research were identified: (1) With the implementation of new technologies, what gaps and biases arise that impact their inclusivity and equity? (2) Although we can assess the immediate and short-term benefits of these technologies, how do we assess their long-term impact on maternal and child health? (3) How do we improve interoperability between different digital platforms to enable seamless data sharing and communication? (4) What policies would need to be put in place to ensure that patient data and privacy are protected? How would this integrate patient consent? (5) How do we understand the needs of the user (patients and health care service providers) and design resources that are user-centered? How do we ensure that users will engage with these resources?

### Patient Engagement Sessions

The 4 members of the PAC varied in languages spoken (1 Mandarin, 1 Cantonese, 1 Spanish, and 1 Arabic), age of their children (1 still pregnant at the start of the project, 1 younger than 6 months, and 2 between 6 months and 1 year), mode of delivery (2 vaginal birth and 2 cesarean section), and location of their birth experience (2 Vancouver Coastal Health, 1 Fraser Health, and 1 Interior Health). Over the course of our 4 group meetings, we completed empathy maps, user journeys (Multimedia Appendix 1), and insight statements and collected reflective notes and recordings of live discussions. Common challenges were experienced among all patient partners navigating the perinatal period. For example, all patient partners described difficulties in understanding the different types of perinatal care providers and how to find an available midwife or doctor in their region. They all also spoke of challenges in navigating the transition from pregnancy to early newborn care, particularly when they had nonurgent concerns about their new baby because many available health care resources focus on urgent and emergency conditions.

In the final PAC meeting, we reflected on the summaries of the ThoughtExchange, MSS report, and BCDHS to identify recurring themes, priorities, and gaps that were also expressed by patients, which were then presented in a master list of insight statements (Textbox 1). These insights were divided into 6 themes: education or health information, navigating health systems, decision support, community or peer support, connected systems, and patient empowerment. Overall, over the 4 meetings, patients clearly expressed that they wanted digital innovation that would support their confidence and ability to navigate the health system in all regions of the province of British Columbia where health care is currently distributed and often fragmented. This should come in the form of free and accessible educational resources and tools to navigate the perinatal health journey. All patient partners discussed how stressful it is to navigate the current system and the frustration of having to communicate their health information to different care providers repeatedly. Almost all of our patient partners moved during the course of their pregnancy and experienced challenges finding maternity care providers both initially and after they moved. Another universal experience was the challenge presented by our current system, which does not allow for the pairing of the mother’s and infant’s health records. This often resulted in logistical challenges in navigating postpartum and postnatal follow-up that were borne entirely by the new parents. As a result of the success of these sessions, the research team worked with the PAC to develop a toolkit for patient engagement that was made available for use by other research teams (Multimedia Appendix 2).

**Final list of insight statements.**

**Reliable health information is needed**

- Patients who are accessing perinatal care are overwhelmed by navigating an abundance of resources, and they are not sure which ones are accurate to follow.
- When seeking health-related information on the internet, information is often conflicting or misleading.
- When seeking health information on the internet, information is only in English and can be difficult to understand or communicate to multilingual family members.
- Health information provided is in technical terms that patients are unfamiliar with leaving patients confused.
- Patients often rely on informal peer support, which adds to the conflicting information they receive, leaving them more confused and unsure of what information to trust.
- Due to the abundance of information available on the internet, patients feel like they are wasting or spending too much of their time filtering through the information.
- Patients indicated that it is challenging to understand the difference between an obstetrician and a midwife especially for newcomers or immigrants from a different health care system; they do not know how to choose the right professional for them.

**Lack of supports**

- Due to the duration between pregnancy and a patient’s first obstetrician gynecologist appointment being too long, patients are left with the responsibility of seeking information independently.
- There are limited perinatal classes that are poorly advertised and are not free; therefore, not all patients have the means to access them.
- Patients are navigating aspects of the health care system during their perinatal journey that they have never had to use before; this often comes with little guidance and doubts about what steps need to be taken.
- Patients who are pregnant for the first time do not know where and what services to seek, leaving them feeling helpless and guilty if complications occur.

**Need for continuum of care**

- Patients’ care is interrupted by the transfer of care to a different care provider especially after delivery. These periods of interruption leave patients uncertain and lost.
- After being discharged after delivery or from midwives, patients feel like their only support is the emergency room, especially if they do not have a family doctor.

**Mental health challenges**

- Complications during the perinatal period leave patients feeling incompetent and highly anxious about their own situations.
- Patients wanting to seek mental health support are discouraged because they are unaware of how to access them or told they are not in need of them.

**Emergency screening**

- Due to the long wait times in the emergency room and lack of knowledge of problems arising either during prepartum or postpartum, patients indicated a need to have an emergency screening process tailored to pregnant and newborn mothers by using the digital platform before they head to the hospitals to save or rule out unnecessary visits.

**Uncoordinated transitions of care**

- It was challenging for patients to find an appropriate obstetrician or midwife in a timely manner; they had to call a list of clinics on their own or find one through word of mouth in order to be accepted.
- When patients find out they are pregnant, they are frustrated by a lack of information on available care providers in their region.
- Patients are confused about when to choose a midwife or other type of doctor and why they may need to switch providers during pregnancy.
- Mom and baby’s care is not coordinated in the health system after the birth of the baby because they are treated as 2 distinct patients.
- There is no standardized system to share health records between perinatal care providers, which leaves patients with the responsibility to communicate their health information when multiple types of caregivers are needed to support their pregnancy.
- When accessing care, services, or resources across different health authorities, health authorities do not follow standardized structures or guidelines acting as an additional barrier for patients as they learn to navigate a new health authority.
- There is a lack of communication and awareness among health authorities, therefore, interrupting patients’ care.

**Poor patient-centered care**

- Care providers are dismissive of patient concerns and thoughts, resulting in patients advocating for themselves and leaving them stressed and isolated.
- Patients feel uninvolved in their health because they are often told what to do without direct and clear explanations or guidelines from their providers.
- Patients have to manage multiple appointments with different providers instead of an integrated care team, meaning that they often wait long periods of time before their needs are addressed.
- Patients would appreciate more visits and opportunities to meet with their care providers or a trusted source in order to discuss their concerns and ask questions.
- Patients want to hear follow-up from their care provider after every test, even if it is normal.

### Final Workshop

**Figure 2:**
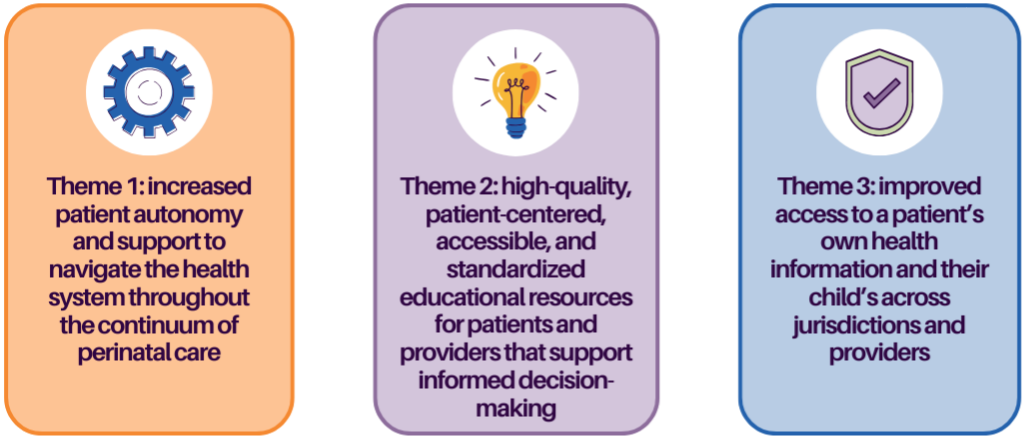
Three priority themes for perinatal digital health research.

**Figure 3:**
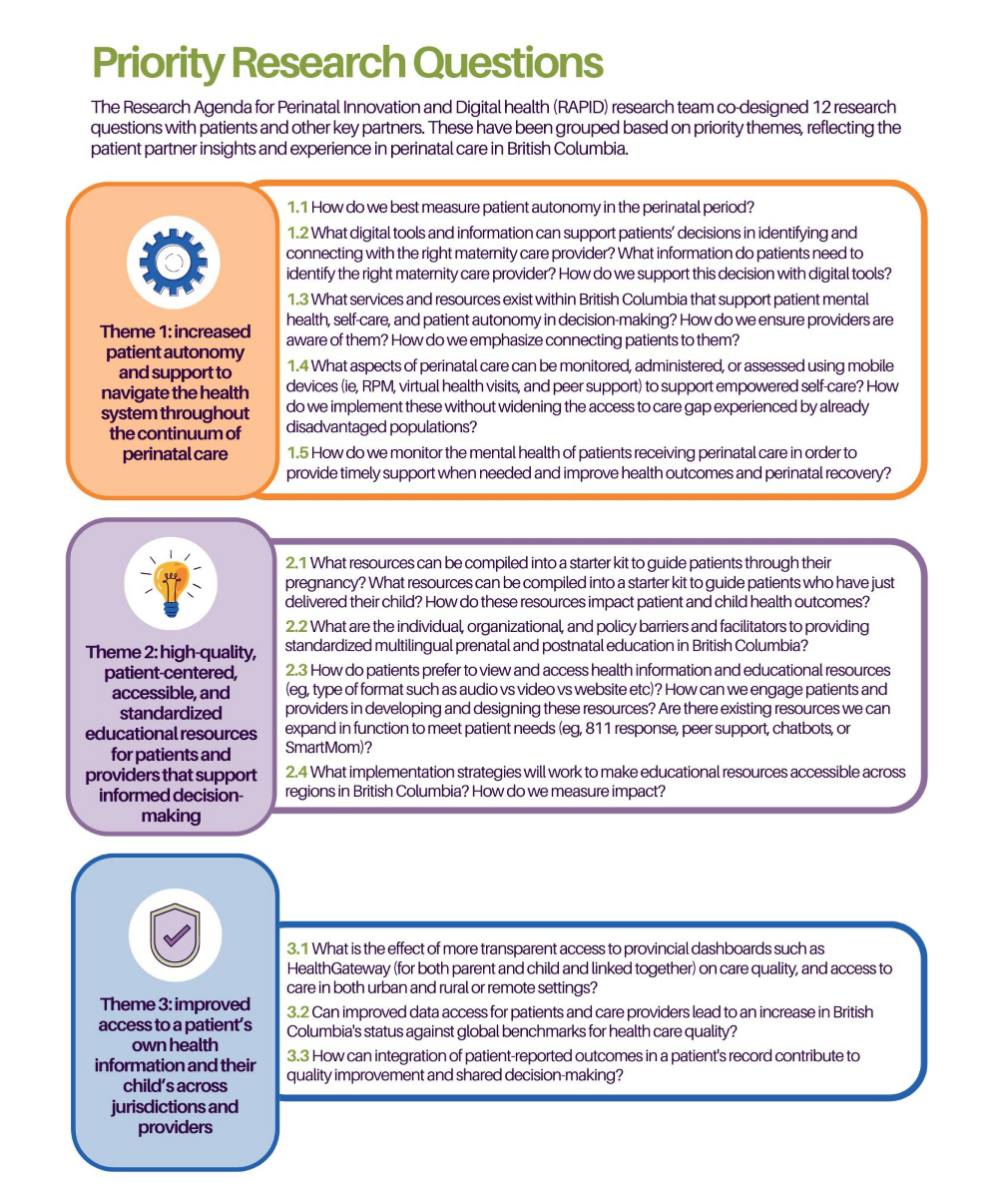
Final list of priority research questions. RPM: remote patient monitoring.

The workshop had a total of 20 attendees: 4 core project team members, 4 patient partners, 2 researchers, 5 clinicians, and 5 health system partners. The workshop was held over Zoom. During the workshop, 3 research themes were chosen as priorities through the discussion process including patient autonomy and support, educational resources for patients and providers, and access to health information (Figure 2). Attendees chose to frame the final research questions around broad transformative goals rather than specific clinical care concepts or processes to make the final agenda broadly applicable across health settings in the British Columbia public health system. For example, although patients and clinicians identified a need for lactation support earlier in the engagement process, a decision was made not to name this clinical concern specifically, as it was felt to narrow the scope of the recommendations. Similarly, patients and care providers identified a need for more accessible support for nonurgent health concerns. Suggestions were made to create more digital resources, but in discussion, the group agreed that more foundational knowledge on the type of digital technology that would be found most usable and effective for providing health information is needed. This resulted in final research questions such as 1.3 and 2.3. A total of 12 final research questions to be included in the research agenda were developed to reflect the identified priorities and patient perspectives (Figure 3).

A follow-up REDCap survey was sent to a total of 35 individuals, including all workshop participants, steering committee members, and patient partners, and we received 20 responses. Respondents included 5 researchers, 5 health system decision makers, and 6 perinatal health care providers with 4 patient advisors. When asked to select the top 3 priority research questions, respondents prioritized questions 1.4, 1.2, and 3.3 (Textbox 2). All of these questions focus on supporting patients’ decision-making with better information and fall within the themes of improving patient autonomy and access to information. When comparing the priority of the overarching themes, overall questions from theme 1: patient autonomy and support were the most selected; however, if we adjust for the number of questions per category, questions from theme 3: access to health information were the most selected.

**Prioritization of research questions.**

**Number of times prioritized: 9**

- Question 1.4: What aspects of perinatal care can be monitored, administered, or assessed using mobile devices (ie, remote patient monitoring, digital health visits, and peer support) to support empowered self-care? How do we implement these without widening the access to care gap experienced by already disadvantaged populations?

**Number of times prioritized: 7**

- Question 1.2: What digital tools and information can support patients’ decisions in identifying and connecting with the right maternity care provider? What information do patients need to identify the right maternity care provider? How do we support this decision with digital tools?
- Question 3.3: How can the integration of patient-reported outcomes in a patient’s record contribute to quality improvement and shared decision-making?

**Number of times prioritized: 5**

- Question 1.5: How do we monitor the mental health patients receiving perinatal care in order to provide timely support when needed and improve health outcomes and perinatal recovery?
- Question 2.1: What resources can be compiled into a starter kit to guide patients through their pregnancy? What resources can be compiled into a starter kit to guide patients who have just delivered their child? How do these resources impact patient and child health outcomes?
- Question 2.3: How do patients prefer to view and access health information and educational resources (eg, type of format such as audio vs video vs website)? How can we engage patients and providers in developing and designing these resources? Are there existing resources we can expand in function to meet patient needs (eg, 811 response, peer support, chatbots, or SmartMom)?
- Question 3.1: What is the effect of more transparent access to provincial dashboards such as HealthGateway (for both parent and child and linked together) on care quality, and access to care in both urban and rural or remote settings?

**Number of times prioritized: 4**

- Question 3.2: Can improved data access for patients and care providers lead to an increase in British Columbia’s status against global benchmarks for health care quality?

**Number of times prioritized: 3**

- Question 2.2: What are the individual, organizational, and policy barriers and facilitators to providing standardized multilingual prenatal and postnatal education in British Columbia?

**Number of times prioritized: 2**

- Question 1.1: How do we best measure patient autonomy in the perinatal period?
- Question 1.3: What services and resources exist within British Columbia that support patient mental health, self-care, and patient autonomy in decision-making? How do we ensure providers are aware of them? How do we emphasize connecting patients to them?

**Number of times prioritized: 1**

- Question 2.4 What implementation strategies will work to make educational resources accessible across regions in British Columbia? How do we measure impact?

## Discussion

### Research Priorities and Implications for Practice

The RAPID project resulted in the co-design of a comprehensive research agenda for perinatal digital innovation that reflects provincial strategies and patient experience in British Columbia. During the project, a series of engagement activities allowed us to ascertain the current gaps and needs for digital health interventions for perinatal care and develop a research agenda that will address these needs. Our research agenda provides a foundation for identifying, developing, and implementing digital solutions that are responsive and feasible within the current British Columbia health care system landscape. The RAPID research agenda identified a core set of research priorities under 3 interdependent thematic areas that, if addressed, could lead to meaningful improvements in patient and clinician experience and health outcomes in the perinatal period. The priority themes identified are patient autonomy and support when navigating the health system, patient and provider access to education resources, and patients’ ability to access health information. These themes align with the BCDHS and have broad support from health system leaders.

The research themes identified and research questions prioritized in this project suggest that further innovation is needed to properly leverage digital health tools to support perinatal care in British Columbia. Priority research questions and a major area of discussion with our patient partners focused on a need for better digital tools to support self-care and decision-making across the entire perinatal period, from pregnancy to the end of the first year of the infant’s life. The ThoughtExchange also highlighted that, within the British Columbian health system, there is a limited number of tools in use that clinicians view as valuable to care. This results in patients accessing numerous external digital resources that are not always of quality and relevance to their care. This agenda can be used to advocate for change at the provincial level and support the incorporation of digital health into the health system. Addressing research priorities identified by RAPID would improve the patient experience by providing needed solutions that allow patients to safely interact with their health care professionals through web-based platforms or through their mobile devices, access self-care tools and applications, use smartphones or other devices to manage and monitor their health, and adopt technology in their health plans.

Digital health innovation as suggested by our research agenda is appealing because it is not restricted by time or place [13] allowing for patient empowerment as well as patient participation in the decision-making of their own health. Addressing the RAPID research priorities can also improve the continuity of care and delivery of essential services, something that all partners in the RAPID project would clearly value. There is already evidence that digital innovation in perinatal care can lead to improved health outcomes for both the woman or pregnant person and their infant [1,12-14]. This agenda highlights that the perceived value of digital innovations for patients receiving perinatal care and their families cannot only be determined by traditional health outcomes. Digital innovations should also be measured against their ability to improve confidence and autonomy in decision-making, self-care, information sharing, and patient and clinical experience.

The digital health sector receives enormous investment, making available a vast number of solutions varying in quality and capabilities [17]. The existing literature has provided a broad understanding of the feasibility, acceptability, and effectiveness of digital tools outlining advantages and disadvantages [1,12]. However, this continued growth in digital solutions does not guarantee or equate to an increase in solutions that can be implemented to meet the actual existing needs of patients, providers, and health systems [17]. In this project, we used HCD methods to identify patient needs and priorities for digital innovation that are aligned with clinical care providers and health system priorities, but our identified priorities cannot exist in isolation from the current knowledge of socioecological factors. Although the RAPID priority research questions are defined based on need, these must be explored with consideration for the facilitators and barriers that contribute to engagement with digital health solutions. Previous research on digital health use has identified barriers and facilitators to implementation such as infrastructure, equipment, network access, motivation, and usability [25,26].

The effort was made to integrate the knowledge of barriers and facilitators to engagement and uptake of digital health innovations throughout the research agenda, such as geographic settings, data-security concerns, and diversity in health concerns. However, considerations for these barriers and facilitators should continue beyond what the questions have defined, as we work to action the agenda. Issues such as connectivity, user motivation, social support, digital literacy, user-friendly design, and credibility should continue to be examined throughout subsequent digital health research.

### Strengths and Limitations

This project’s use of a human-centered approach prioritized patient voices and provided the space for collaborative co-design of the final research questions. Limiting the number of patient partners and restricting the number of researchers in the patient advisory meetings allowed for better quality engagement through intimate and extensive discussions. The diversity of partners from across British Columbia allows for more generalizable results. However, a significant limitation was our lack of engagement with Indigenous clinical care providers or patients. There is a need to engage with Indigenous people’s voices within all of the partner groups before moving research forward from this strategy. Related to this, although we were able to collect a diverse group of partners’ opinions and bring these together to design a comprehensive research agenda, running the project under a knowledge translation initiative restricted the amount of data we could collect on engaged participants. As a result, it may be more difficult for others to apply this knowledge outside of the British Columbia health system. We also cannot explore if participant characteristics such as age or experience level (for health care providers) had any impact on their opinions of digital health priorities. An additional strength is our engagement with health system partners throughout the project, aligning in strategy and garnering early support from decision makers. As a result, outcomes of research informed by this agenda are more likely to be implemented and achieve long-term impact. There was strong collaboration throughout the project between researchers and knowledge users, allowing us to lead this work within the health system, leveraging developed partnerships, and articulating alignment with organizational goals and patient research priorities.

This HCD approach prioritized patient voices in developing research priorities; yet, our multipartner codevelopment approach recognizes that the ownness should not be on patients alone to identify a solution to the complex issues of perinatal health care delivery. The emphasis on people solving their own problems that is core to the HCD approach cannot be directly assumed in a health care context due to the complexity of the system and the multitude of interested parties that will be impacted by any system changes. In positioning our work to address patient needs while still aligning with health system priorities, we have aimed to support value-based care efforts in the British Columbia health system. Focusing further research and innovation efforts to address the RAPID research priorities can support improvements in value-based care, as they would improve outcomes and processes that matter to patients without an unreasonable increase in health care expenditure.

### Conclusions

This project identified clear support and enthusiasm across partners for digital innovation to improve the quality of perinatal care in the province of British Columbia. Our research agenda highlights the need for better patient education and health information systems that are more accessible to patients and care providers with the overarching need for digital resources that can increase patient autonomy in decision-making throughout pregnancy and the transition to newborn care. Co-design of this research agenda is a first step in the research process. Our priority now is to take action on identified research priorities. Action can occur through traditional research funding pathways but also by advocating to our health system partners to directly address needs identified by the RAPID research agenda within the health system.

## Appendix

Multimedia Appendix 1 Anonymized example of a user journey.

Multimedia Appendix 2 Toolkit for patient engagement.

## Abbreviations

BCDHS: BC Digital Health Strategy
HCD: human-centered design
MoH: Ministry of Health
MSS: Maternal Services Strategy
PAC: patient advisory committee
PHSA: Provincial Health Services Authority
PSBC: Perinatal Services BC
RAPID: Research Agenda for Perinatal Innovation and Digital Health
REDCap: Research Electronic Data Capture
