# Supplementary figures and images for: The Research Agenda for Perinatal Innovation and Digital Health Project: Human-Centered Approach to Multipartner Research Agenda Codevelopment
Source: JMIR Hum Factors. 2025 Jan 30;12:e60825. doi: 10.2196/60825 (PMC11826941; doi:10.2196/60825)

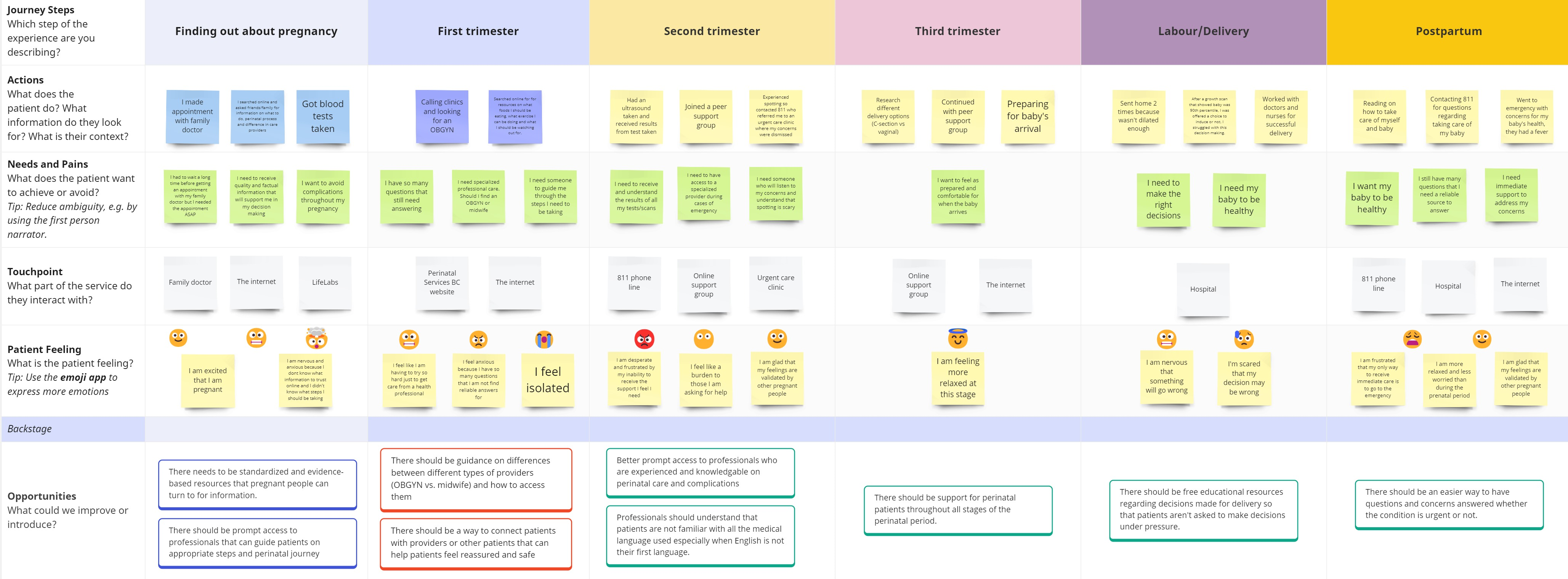

Supplement: Multimedia Appendix 1 [file humanfactors_v12i1e60825_app1.png]
