# Supplementary material for: The Research Agenda for Perinatal Innovation and Digital Health Project: Human-Centered Approach to Multipartner Research Agenda Codevelopment
Source: JMIR Hum Factors. 2025 Jan 30;12:e60825. doi: 10.2196/60825 (PMC11826941; doi:10.2196/60825)
Supplement: Multimedia Appendix 2 [file humanfactors_v12i1e60825_app2.pdf]

Engaging with Patient Partners:

A DIGITAL HEALTH RESEARCH APPROACH

2023

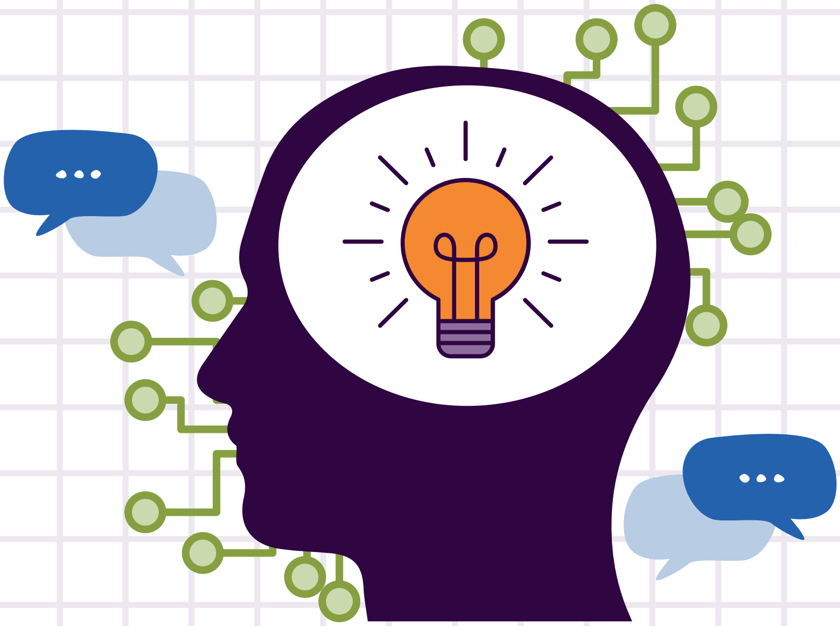

WOMEN'S HEALTH RESEARCH INSTITUTE AT BC WOMEN'S

BC Children's Hospital Research Institute

ACKNOWLEDGEMENTS

This Toolkit was prepared by Haneen Amhaz, Sally Chen and Beth Payne with comments and input from the RAPID Steering Committee, Tina Costa, Kathryn Dewar, Quynh Doan, Kathryn Berry-Einarson, Gina Ogilvie, Nicole Prestley, Tibor van Rooij, May Tuason, Marianne Vidler. This project was funded by Michael Smith Health Research BC. More importantly, this Toolkit could not exist without our patient partners: Amanee Elchehimi, Kylin Han, Jade Morales, and Lu Yao for which we are grateful.

This Toolkit draws heavily from the following material:
The Field Guide to human-centered design. 1st ed. San Francisco: IDEO.org; 2015.

Please cite the work as follows:
Women's Health Research Institute. (2023). *Engaging with Patient Partners: A Digital Health Research Approach*. Vancouver, BC.

For more information on the RAPID project please visit: https://whri.org/our-initiatives/rapid/

THE APPROACH

What is it?

The following is a toolkit of resources for engaging patient partners in digital health research. This approach is based on the methodology used by the Research Agenda for Perinatal Innovation & Digital health (RAPID) research project.

Defining the scope of the approach

This strategy is informed by design thinking and user experience research methods in order to facilitate interactive discussions and sharing of experiences while limiting the room for bias. These methods create a space where patients feel empowered to share their thoughts, experiences, and needs. Utilizing design thinking and user experience research approaches allows for the amplification of patient voices and places patients at the center of the research.

The human-centered design has 3 core components:

1. **Participatory co-design** so those who will use or be impacted by your design are involved with the research team in a meaningful way.
2. **Enhancing human abilities** to empower those who will use it.
3. **Incorporating human values into an iterative approach** where there is genuine interest in the whole person and their circumstances are considered in tandem with technological requirements.[1]

This iterative approach consists of 5 principles[2]:

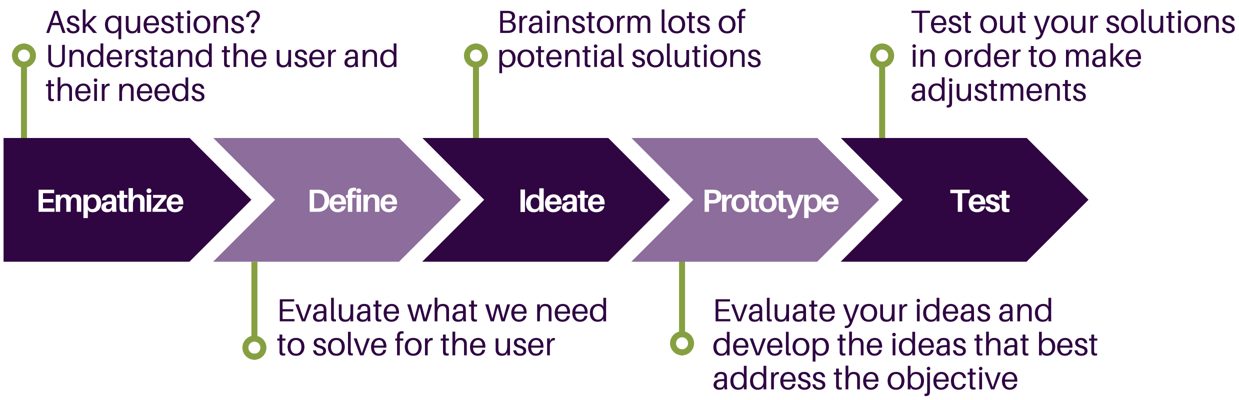

Target audience

This toolkit is intended for researchers working in the field of digital health innovation. Patients are essential partners in the research process due to the real-world experiences, perspectives, and insights that they can contribute. This enhances the relevance of outcomes ensuring they are appropriate and address patients' genuine concerns.

How to use it

Below you will find an outline for four sessions. Each session includes an objective, a pre-meeting activity (except Session One), and activities for a 1-hour virtual synchronous meeting. These sessions were designed with the following considerations in mind: length of meetings, frequency of meetings, mode of delivery, and synchronicity of activities.

Important Factors to Consider

NOTE: All of these factors should be discussed with your patient partners at the start of the project.

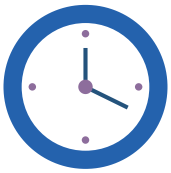

Length of Meetings

It is important to consider how much time your patient partners can dedicate to a synchronous meeting. You also need to acknowledge that there is sufficient time for partners to share their voices and go through activ to share their voices and go through activities.ities.

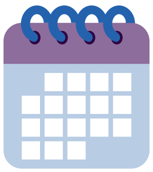

Frequency of meetings

Patient partners have other commitments so recognizing how often patients can meet up can ensure patients remain engaged.

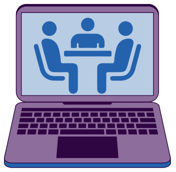

Mode of delivery

Patients have different preferences. If you are wanting to capture perspectives from wide geographic areas, virtual options for delivery may be ideal.

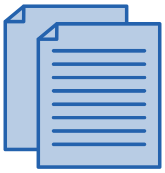

Synchroneity of activities

Considering patients have different commitments and arranging multiple and lengthy meetings can be difficult, some activities can be done asynchronously at a patient's convenience.

Although the activities are designed to gain insight into patient experiences, you as a researcher should also be completing and contributing to the conversation when able to. This creates a power balance where patients can start to view themselves as partners in your research rather than subjects of your research.

In order to adapt this toolkit you should reflect on these considerations and determine what would work best for your desired research outcomes and patient partners. It is therefore important to consult with your patient partners to understand their needs and capabilities. Tailoring your plans to your patient partners will allow for more engagement.

OVERALL SCHEDULE

Session Number	Objective	Pre-session Activity	Session Activities
1	Establish an environment where patients feel safe and comfortable sharing their experiences		Ice breaker<br>Introduce project
2	Gather patients' perspectives and experiences in the healthcare system	Empathy map<br>30-minute reflection	Share empathy maps and experiences
3	Identify touchpoints and potential areas with opportunities for improvement in the healthcare experience	User journey<br>Reflection on fellow patients' experiences	Discussing user journey
4	Compile insights from experiences and meetings	Insight statement worksheet	Go over insights

A more detailed breakdown of each week follows. Templates for all activities are included with each week and can be found at the end of the toolkit.

WEEK ONE

Objective:
Establish an environment where patients feel safe and comfortable sharing their experiences

Synchronous Activity During Session

Icebreaker

Starting your patient engagement with an icebreaker is an important step in encouraging participation and allowing patient partners to feel more comfortable with each other and the research team.

Introductions
You should start with unintrusive questions that help everyone get to know each other. The research team should also answer all questions to show equal participation

Information you could ask for include name, favorite food, favorite book, favorite movie, fun fact, etc.

You can also tailor these based on who your patients are. For example, if you are engaging with multilingual patients you can ask them to share what languages they speak.

This should take the majority of the meeting as it is an essential part of setting the pace for the rest of your meetings.

Conversation Starter
To encourage and begin thoughtful reflection and discussion into your research question, start by asking a broad question related to your topic.

A Mentimeter word cloud is a great tool for this. Asking patients to share the words that come to mind when they think of your topic prepares patients for future comprehensive discussions.
Link to Mentimeter: https://shorturl.at/CEFT8

WEEK TWO

Objective:
Gather patients' perspectives and experiences in the healthcare system

Pre-Session Activity

Empathy Mapping

Empathy mapping is a simple visual engagement tool that allows patients to share their first-hand thoughts, preferences, emotions, and challenges during a single experience in their journey. This map focuses on only one event. It acts as a starting step in unpacking their entire healthcare experience.

To start ask patients to write out one memorable event from their healthcare experience. Then have them reflect and fill out the following information (see Appendix A):

- *Says/see:* What do they verbalize and share? What did they observe? What were they watching and reading?
- *Thinks:* What did they think about their experience at the time it occured? What occupies their thoughts?
- *Does:* What actions did they take? How did they do it?
- *Feels:* What was their emotional state? How did they feel about the experience?
- *Pains:* Any frustrations, challenges, and/or obstacles that prevented them from achieving their goal.
- *Gains:* Their needs. The benefits that they would have experienced if the "pains" were removed.

For more information on empathy mapping: https://shorturl.at/gtEV2

Reflection

A 30-minute reflection allows patients to reflect more broadly on their experience and put it into words. This allows them to prepare for future activities as well as allows for the research team and other patient partners to make connections with the experiences. Allow patients to reflect however they feel comfortable, for example through paragraphs or bullet points, as well as providing prompts to guide them (see Appendix B).

WEEK TWO

Synchronous Activity During Session

Sharing Circle

Give patients the opportunity to present their empathy maps as this allows for an accurate interpretation of their map. Also provide them with time to go beyond the map and touch upon what stood out to them when writing their reflections.

This is a great opportunity to encourage patients to start making mental connections with each other's experiences.

It is important to reiterate that the research team present at these meetings should also have an empathy map and reflection complete if they have experienced this health condition. If not research team should avoid interpreting the patient experiences and focus on active listening while letting the patients lead. As meeting facilitators, the researcher team should be asking open-ended questions, not make assumptions, validate patients feelinlgs. Check in with the patient partners to ensure that you understood what it is they are trying to communicate as this is the purpose of having live discussions.

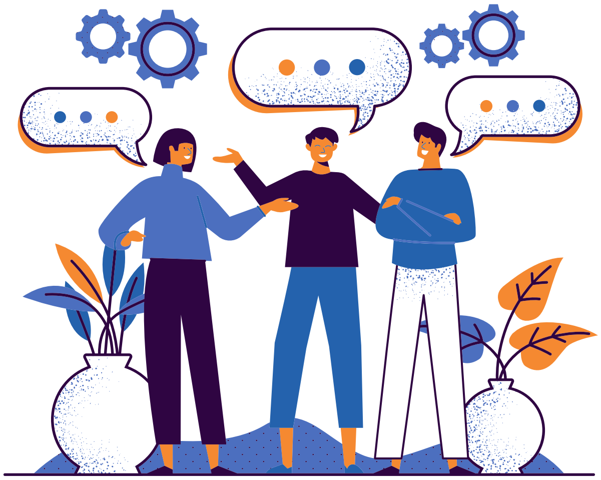

WEEK THREE

Objective:
Identify touchpoints and potential areas with opportunities for improvement in the healthcare experience

Pre-session Activity

User Journey

The empathy map allowed patients to analyze and focus in on a singular experience, the user journey now allows them to reflect on all their experiences.

A user journey is a diagram that represents a patient's entire interaction with the system during the particular period you are interested in. It breaks down their journey into a sequence of steps/stages during the period of interest, asking what patients were doing, thinking, feeling, and experiencing at each step/stage. User journeys are a great tool for allowing researchers to recognize the gaps and areas for opportunities informed by patients needs and how they interact with the healthcare system.

You can either divide the period of interest into stages you feel to be appropriate or allow patients to do this themselves (see Appendix C). For each stage have patients reflect and take note of the following aspects:

- *Actions:* What did they do? Include the context, so why or how? What information were they looking for?
- *Needs and Pains:* What they were trying to achieve? What prevented them from achieving it? What was a positive outcome (green sticky notes)? What was a negative outcome (pink sticky notes)?
- *Touchpoints:* What parts of the healthcare system they interacted with? What services did they use? What resources did they rely on?
- *Patient Feeling:* How did they feel during that time?
- *Opportunities:* What improvements can be made based on their experience? What can be introduced?

For more information on user journeys: https://shorturl.at/fglxz

WEEK THREE

Synchronous Activity During Session

Interactive Discussion

Patients should go over key aspects of their user journey. Other patients should be encouraged to comment on each other's experiences and share their thoughts verbally or through the Zoom chat box if virtual. This will allow the patients to create connections with each others experiences, creating rich discussion around the research topic.

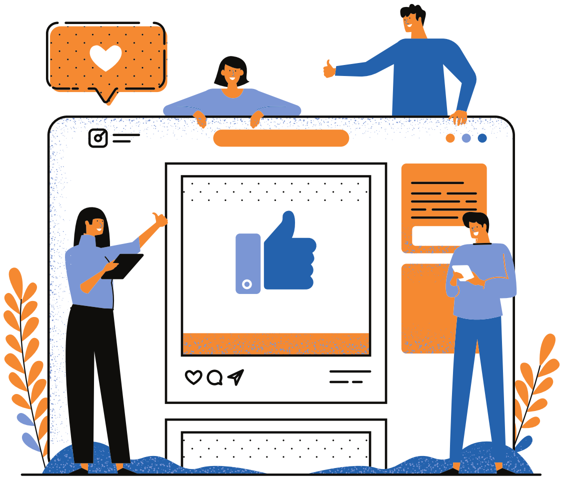

WEEK FOUR

Objective:
Identify problem statements

Pre-session Activity

Insightful Statements

After reflecting on their personal experience and the experiences of each other, patients should now be able to identify insight statements.

Insight statements are findings or observations that answer the core question that is driving the research. Patients should first identify what the overarching themes have been throughout the discussion then identify the challenges within these themes and turn them into 2 to 3 sentences (see Appendix D). These sentences put a single gap, barrier or observation into concise, specific and focused words. Insight statements should provide an understanding of the problem while identifying a single pain point, who the pain point is for and what their experiences are.

For example, patients may have found lack of reliable information to be a theme. Within that theme an insight could be: *when seeking health-related information online, information is often conflicting or misleading leaving perinatal patients confused.*
For more information on insight statements: https://shorturl.at/cxN26

Synchronous Activity

Interactive Discussion

Patients should take turn sharing the insight statements they felt were of priority. This should be an open discussion for all patients to discuss and reflect on each others statements to identify common insights.

CONCLUSION

This series of activities was designed to build upon each other to provide a clear and holistic understanding of patient experiences. These methods of design thinking research are particularly beneficial in digital health research because they acknowledge that effective digital health innovation needs to be responsive to the actual needs of its users.

Important Takeaways

This toolkit is meant to be a guide for your patient engagement strategy and can be adapted to better suit your audience. However, there are key aspects of this toolkit that you should adhere to:

1. Members of the research team should participate in all planned activities when possible and and also focus on being active listeners because this will help patients feel more comfortable sharing. It also acknowledges that the patients are partners in your research.
2. Researchers should be open to hearing new ideas and solutions that they may not have expected. A core principle of conducting human-centered design is empathy which emphasizes patient leading the discussion.
3. Having a session dedicated to allowing all patients and researchers to familiarize themselves with each other. This step is crucial to ensuring the following meetings are fruitful as it allows for everyone to be comfortable sharing honestly with each other.
4. Allow patients the time to share and discuss all the activities they did. This always for a more accurate understanding of their experiences as opposed to simply having the research team read over the completed activities.
5. Be open to making adjustments as your meetings progress to accommodate your patient partner's needs and preferences.

You can find templates for all the activities in the following Appendix.

Suggestions

When conducting the activities the RAPID research team utilized Miro for all the activities. Miro is an online digital collaborative workspace. This allowed all patients and researchers to view everyone's completed activities as well as leave comments. This is also convenient during the synchronous session because it allows for everyone to view the single workspace with everyone's activity during the discussion.

REFERENCES

1. Evans L, Evans J, Pagliari C, Källander K. Scoping review: Exploring the equity impact of current Digital Health Design Practices. Oxford Open Digital Health [Internet]. 2023 [cited 2023 Sept];1. Available from: https://doi.org/10.1093/oodh/oqad006
2. Design Thinking [Internet]. Interaction Design Foundation; [cited 2023 Sept]. Available from: https://www.interaction-design.org/literature/topics/design-thinking#:~:text=Design%20thinking%20is%20a%20non,are%20ill%2Ddefined%20or%20unknown.

ADDITIONAL RESOURCES

The Field Guide to Human-Centered Design **by IDEO.org**
https://www.designkit.org/resources/1.html
A free book that explains the human centered design process and includes activity suggestions for all the various steps. The activities in our toolkit were adapted from this book.

Scoping review: exploring the equity impact of current digital health design practices
https://academic.oup.com/oodh/article/doi/10.1093/oodh/oqad006/7197197
An article that discusses the core components of human centered design in the context of digital health.

Innovating health care: key characteristics of human-centered design
https://www.ncbi.nlm.nih.gov/pmc/articles/PMC7802070/
Provides a deeper understanding of the human-centered design process and how it can be applied within healthcare and research.

EMPATHY MAP

Reflect on a memorable instance from your healthcare experience and take note of what you saw/said, thought, did, and heard as well as any pains or gains.

A memory that stand out to me is ...

Name

Says/Sees

Thinks

Does

Feels

Pains

Gains

REFLECTION

Reflect on your healthcare experience to gain a deeper insight into your journey. Express any thoughts or experiences that you feel are meaningful such as events, milestones, emotions, feelings, concerns, challenges or fears. This does not have to be in formal paragraph form; write in whichever way aligns with your reflection process.

USER JOURNEY

Divide your journey into stages then fill out the following chart using the provided prompts.

**Steps**			
**Actions**<br>What are you doing? What information do you look for?			
**Needs and Pains**<br>What do the you want to achieve or avoid?			
**Touchpoint**<br>What service did you interact with?			
**Patient Feeling**<br>What are you feeling?			
**Opportunities**<br>What could we improve or introduce?			

INSIGHT STATEMENTS

Reflect on your experiences, the completed activities, and the group discussions to identify overarching themes. Then within these themes write 2-3 statements that communicate a problem while identifying a single pain point, who the pain point is for and what their experiences is.

Theme:

Insight Statements:

1.

2.

3.

Theme:

Insight Statements:

1.

2.

3.

Theme:

Insight Statements:

1.

2.

3.
